# Nail-Patella Syndrome Associated with Short Stature: A Case Series

**DOI:** 10.1155/2010/869470

**Published:** 2010-08-08

**Authors:** Samir Haddad, Leila Ghedira-Besbes, Chahra Bouafsoun, Sabeur Hammami, Slaheddine Chouchene, Chebil Ben Meriem, Mohamed-néji Guediche

**Affiliations:** Department of Paediatrics, CHU Fattouma Bourguiba, 5000 Monastir, Tunisia

## Abstract

*Introduction*. Nail-patella syndrome (NPS) is a rare genetic disorder that is characterized by a pleiotropic malformation affecting the nail, the skeleton, and occasionally the central nervous system and the kidneys. *Case Presentation*. We report two paediatric cases, which are of two sisters, who aged, respectively, two and five years. They are admitted to explore short stature. The initial clinical examination and radiologic findings confirmed the diagnosis of Nail-patella syndrome. *Conclusion*. Skeletal, ophthalmologic, and renal involvements were mostly associated with NPS. The association with short stature was exceptional.

## 1. Introduction

Nail-patella syndrome (NPS), also known as hereditary osteoonychodysplasia (HOOD) or Turner-Kieser syndrome, is a rare clinical entity with an incidence ranging from 4,5 to 22 per million [1, 2]. It is an autosomal dominant disorder. Both sexes are affected in equal numbers. The NPS is due to mutations in the *LMX1B gene* chromosome 9q34.1 and is linked to the ABO blood group locus [3–5]. NPS is characterised by a clinical tetrad including finger nail dysplasia, hypoplastic or absent patellae, dislocation of the radial head, and bony protuberances of the iliac known as the iliac horns [1, 2]. In addition, soft-tissue abnormalities such as renal dysplasia, muscle weakness, impaired hearing, waddling gate, and scapular winging may be present. Few cases of NPS associated with short stature are reported in the literature [3]. We report two paediatric cases with NPS and short stature.

## 2. Cases Reports

**Figure 1:**
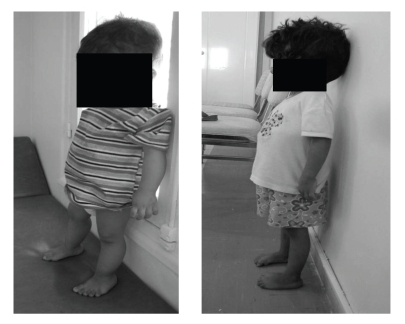
The short stature is evident for the two girls.

H. S. and H. A. are two sisters, who aged, respectively, two and five years. They were hospitalised to explore short stature. Their birth history is normal. Physical examination revealed a short stature (−4 DS) (Figure 1) with no dysmorphic features. 

**Figure 2:**
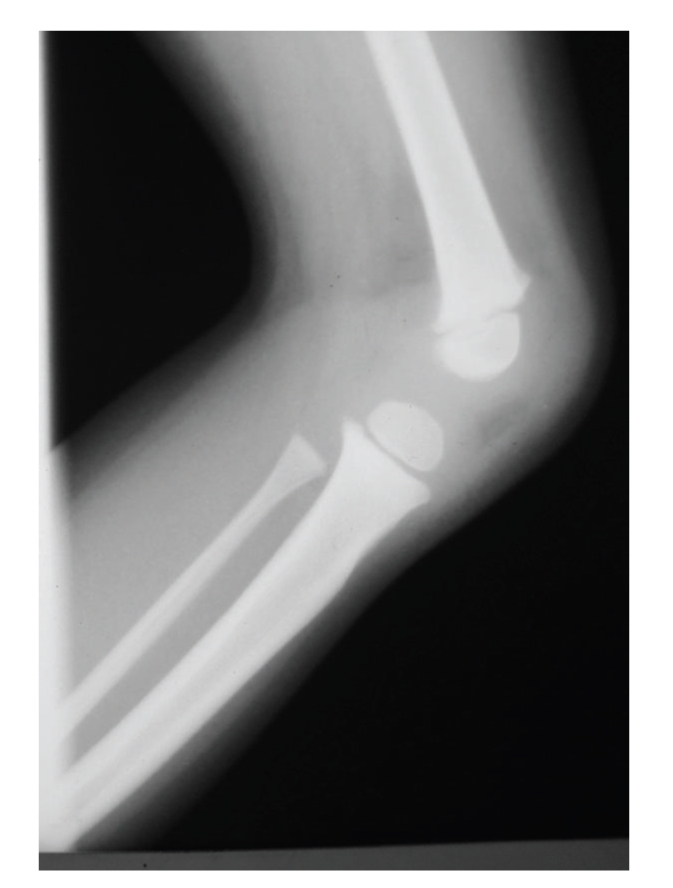
Knee lateral radiographs revealed absence of the patella.

Their upper extremities were thin. Finger nails were flat and grooved, mostly affecting the thumb and diminishing in severity toward the ulnar side of the hand. The toe nails were not involved. Both elbows enjoyed full range of motion without crepitance, including full pronation and supination despite cubitus valgus suggestive of radial head hypoplasia. Further examination of the lower extremities showed equal leg lengths; quadriceps muscles were not well developed and squared at knees with absence of patellae. There is not ligamentous laxity. They had valgus configuration of feet. Knee radiographs revealed bilateral complete absence of the patella with mildly hypoplastic lateral femoral condoyle (Figure 2). 

**Figure 3:**
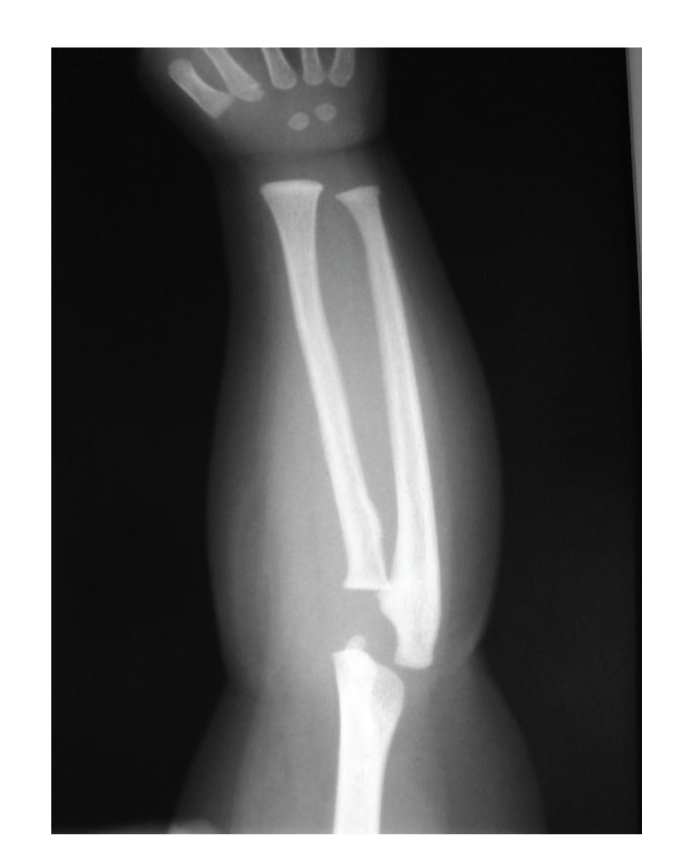
Hypoplasia and subluxation of the head of the radius.

Radiographic evaluation also showed hypoplastic radial head (Figure 3) and the absence of iliac horns.

The diagnosis of NPS was established on basis of the the characteristic constellation of physical and radiologic findings. Subsequently, a full renal and ophthalmologic workup was performed to investigate the presence of any abnormalities. No proteinuria or hematuria was present, and renal ultrasound examination was normal. The ophthalmologic examination was unremarkable. With basic growth hormone dosage and after stimulation with insulin, thyroid hormone dosage and celiac serology were normal.

## 3. Discussion

Hereditary osteoonychodysostose is frequently used as synonym for the NPS. It indicates the major areas of involvement. Despite complete penetrance, this syndrome exhibits extremely variable expression, not only of the components of the “tetrad” but also of a great number of other facultative anomalies which may affect the central nervous system, the eyes, and the kidneys [2, 4].

A great number of studies were published in the nineteenth century dealing with sporadic and familial cases of congenital absence, delayed development, or subluxation of the patellae; in the retrospect, several of these can be identified as the Nail-patella syndrome.

The hereditary nature of NPS was recognized more than 70 years ago; identification of the responsible gene was achieved only recently. Targeted disruption of the LMX1B gene in mice results in a phenotype characterized by nail aplasia, absence of patellae, and renal involvement, similar to the human NPS. In human, mutations of the LMX1B gene on chromosome 9 were the third autosomal mutation found to be closely linked to another gene, namely, the ABO locus [3, 4, 6]. Despite the aberrations of a single gene responsible of NPS, the manifestations of the syndrome show marked variability between and even within affected families. Our patients had a severe form of skeletal involvement without renal abnormalities. 

The nail dysplasia may produce a triangular lunula, especially of the index and/or middle fingers. In most cases there is moderately severe hypoplasia of the medial and distal aspects of index and thumbnails. Other modifications of the nails may include narrowness, smallness, spooning, rarely thickening, a median groove or cleft, roughness, cracking, splitting, and/or brownish discoloration with distal fraying. Manifestations of patellae dysplasia range from complete agenesis to moderate hypoplasia; some patients have normal-appearing knee joints. The patella is frequently tripartite or polygonal, and owing to hypoplasia of the patellae tendons. It has a tendency to dislocate laterally, producing a considerate gait impediment especially upon walking downstairs [1, 2].

**Elbow Dysplasia**

The radial head may be small and subluxed dorsally, leading to mild-to-moderately severe impairment of extension, pronation and supination of the forearm, and varying degrees of webbing of the antecubital fossa. The iliac horns are one of the most characteristic but harmless features of the NPS. They arise from the posterior aspect of the ilium and can occasionally be palpated in the substance of the glutei [4].

Other anomalies encountered in affected individuals with the NPS include narrowness and deformities of the sternum, frequent spina bifida occulta, clinodactyly of the fifth fingers, hypothyroidism and goiters, mental retardation, keratoconus, microcornea, microphakia, and cataracts. Renal dysplasia appears to be an infrequent but potentially fatal complication of the NPS.

## 4. Conclusion

The NPS is a pleiotropic malformation syndrome affecting mesodermal and ectodermal derivatives. Skeletal, ophthalmologic, and renal involvements were mostly found. The association with short stature was exceptional.

## References

[1] Brixey AM, Burke RM (1950). Arthro-onychodysplasia. Hereditary syndrome involving deformity of head of radius, absence of patellas, posterior iliac spurs, dystrophy of finger nails. *The American Journal of Medicine*.

[2] Schulz-Butulis BA, Welch MD, Norton SA (2003). Nail-patella syndrome. *Journal of the American Academy of Dermatology*.

[3] Finsterer J, Stöllberger C, Steger C, Cozzarini W (2007). Complete heart block associated with noncompaction, nail-patella syndrome, and mitochondrial myopathy. *Journal of Electrocardiology*.

[4] Lucas GL, Opitz JM, Wiffler C (1966). The nail-patella syndrome: clinical and genetic aspects of 5 kindreds with 38 affected family members. *The Journal of Pediatrics*.

[5] Millá E, Hernan I, Gamundi MJ, Martínez-Gimeno M, Carballo M (2007). Novel *LMX1B* mutation in familial nail-patella syndrome with variable expression to open angle glaucoma. *Molecular Vision*.

[6] Sato U, Kitanaka S, Sekine T, Takahashi S, Ashida A, Igarashi T (2005). Functional characterization of *LMX1B* mutations associated with nail-patella syndrome. *Pediatric Research*.

